# Circulation and Evolution of SARS-CoV-2 in India: Let the Data Speak

**DOI:** 10.3390/v13112238

**Published:** 2021-11-08

**Authors:** Sanket Limaye, Sunitha M. Kasibhatla, Mukund Ramtirthkar, Meenal Kinikar, Mohan M. Kale, Urmila Kulkarni-Kale

**Affiliations:** 1 Bioinformatics Centre, Savitribai Phule Pune University (Formerly University of Pune), Pune 411007, India; sanket.limaye22@gmail.com (S.L.); smanjari@gmail.com (S.M.K.); meenalkinikar@gmail.com (M.K.); 2 HPC-Medical & Bioinformatics Applications Group, Centre for Development of Advanced Computing, Pune 411008, India; 3 Department of Statistics, Savitribai Phule Pune University (Formerly University of Pune), Pune 411007, India; ramtirthkarmr@gmail.com (M.R.); mmkale@unipune.ac.in (M.M.K.)

**Keywords:** COVID-19, SARS-CoV-2, lineage diversity, evolutionary dynamics, virus bioinformatics, India, variant of concern, variant of interest, spike, phylogeny

## Abstract

The COVID-19 pandemic is a global challenge that impacted 200+ countries. India ranks in the second and third positions in terms of number of reported cases and deaths. Being a populous country with densely packed cities, SARS-CoV-2 spread exponentially. India sequenced ≈0.14% isolates from confirmed cases for pandemic surveillance and contributed ≈1.58% of complete genomes sequenced globally. This study was designed to map the circulating lineage diversity and to understand the evolution of SARS-CoV-2 in India using comparative genomics and population genetics approaches. Despite varied sequencing coverage across Indian States and Union Territories, isolates belonging to variants of concern (VoC) and variants of interest (VoI) circulated, persisted, and diversified during the first seventeen months of the pandemic. Delta and Kappa lineages emerged in India and spread globally. The phylogenetic tree shows lineage-wise monophyletic clusters of VoCs/VoIs and diversified tree topologies for non-VoC/VoI lineages designated as ‘Others’ in this study. Evolutionary dynamics analyses substantiate a lack of spatio-temporal clustering, which is indicative of multiple global and local introductions. Sites under positive selection and significant variations in spike protein corroborate with the constellation of mutations to be monitored for VoC/VoI as well as substitutions that are characteristic of functions with implications in virus–host interactions, differential glycosylation, immune evasion, and escape from neutralization.

## 1. Introduction 

The COVID-19 (coronavirus disease 2019) pandemic has spread around the world, impacting 200+ countries. As of 30 August 2021, there are 230,418,451 reported cases and 4,559,229 deaths globally (WHO reference: https://covid19.who.int/; Last accessed 30 October 2021). With 32,695,030 cases and 440,533 deaths, India is the second worst hit country (next to the USA) for number of reported cases and ranks at the third position in terms of number of deaths (next to the USA and Brazil). 

Ever since detection of the first case of COVID-19 on 31 December 2019 in Wuhan, China, the virus has spread all over the world (WHO reference: https://www.who.int/emergencies/disease-outbreak-news/item/2020-DON229; Last accessed 30 October 2021). The first confirmed case of COVID-19 in India was reported on 27 January 2020 from Kerala [1]. Reports of multiple introductions of the virus due to global travel are published [2,3,4]. Being a populous country with densely packed cities, cases of COVID-19 increased exponentially in India in 2020. The respiratory route of virus transmission, high reproductive number, and superspreading events were responsible for its spread to rural communities and amongst all age groups. Two waves corresponding to an upsurge of cases have been reported in India during 2020 and 2021 (https://www.mohfw.gov.in/; Last accessed 30 October 2021). 

The SARS-CoV-2 nomenclature has evolved during the course of the pandemic. GISAID (https://www.gisaid.org/; Last accessed 30 October 2021) [5], NextStrain (https://nextstrain.org/ncov/gisaid/global; Last accessed 30 October 2021) [6], and PANGOLIN (Phylogenetic Assignment of Named Global Outbreak LINeages; https://cov-lineages.org/resources/pangolin.html; Last accessed 30 October 2021) [7] proposed unique lineage nomenclatures. Subsequently, the WHO (https://www.who.int/en/activities/tracking-SARS-CoV-2-variants/; Last accessed 30 October 2021) designated lineage labels such as variants of concern (VoC: Alpha, Beta, Gamma and Delta) and variants of interest (VoI: Eta, Iota, Kappa, Lambda and Mu) based on considerations such as prevalence, transmissibility, detrimental change in epidemiology, increase in virulence, change in clinical disease presentation, and decrease in the effectiveness of available diagnostics, vaccines, and therapeutics (as of 1 September 2021). Assignments of VoC and VoI are also being reviewed periodically by the WHO and CDC (https://www.cdc.gov/coronavirus/2019-ncov/variants/variant-info.html; Last accessed 30 October 2021). All the four nomenclature systems for SARS-CoV-2 are dynamic and are updated to accommodate an expanse of diversity over a period of time. 

In order to characterize SARS-CoV-2 in various parts of India and to understand lineage diversity and distribution, genome sequencing has been carried out since the onset of the pandemic [8,9,10,11,12]. Several genome sequencing studies were performed to characterize SARS-CoV-2 isolates sampled in various States and Union Territories (UTs) of India. The predominance of the B.1.1.7 (Alpha) lineage was detected amidst the presence of multiple other lineages in the state of Karnataka [13], and symptomatic cases have been attributed to the spread in the state at the beginning of the pandemic [14]. Genomic sequencing and haplotype analysis revealed three major introductions of SARS-CoV-2 in the state of Kerala, which were followed by multiple outbreaks leading to local spread [11]. The predominance of lineages 20A and 20D in the state of Gujarat with the nucleotide substitutions C28854T in N-gene and G25563T in Orf3a were observed to be prominent in deceased patients [15]. The mutational landscape of SARS-CoV-2 isolates was characterized from 200 genomes sampled from the southern state of Telangana during March–July 2020, which helped identify mutations in non-structural proteins and revealed the predominance of the 20B lineage [10]. The state of Maharashtra reported the prevalence of B.1.1.7 (Alpha), B.1.617.1 (Kappa), and emergence of B.1.617.2 (Delta), which outcompeted other circulating lineages [16]. Evidence of re-infections by lineages 19A, 20A, and 20B have been reported in Maharashtra [17]. The national capital and UT of Delhi reported several outbreaks in 2020 and 2021 [3]. The Delta variant displaced Alpha and all other variants due to its high transmissibility and immune evasion, thus causing reinfections [12,18]. 

Genomes of SARS-CoV-2 isolates sequenced in India are deposited in the global archives, GISAID [5], and GenBank [19]. The results of genomic characterization were used to understand the virus evolution, lineage diversification, antigenic variation, and characterization of emerging mutations in various genomic regions [10,20,21]. Genome sequencing activities initiated by various laboratories funded by the national and state government agencies were followed by the institution of Indian SARS-CoV-2 Consortium on genomics (INSACOG) funded by the Government of India (https://dbtindia.gov.in/insacog; Last accessed 30 October 2021). 

The majority of studies reported from India pertain to the sequencing of regional and/or national isolates and their downstream analysis. This study is an effort to consolidate the genetic diversity of SARS-CoV-2 across India using data driven approaches. This study is designed to systematically analyze variants of concern (VoC) and variants of interest (VoI), circulating in India with reference to sequencing coverage, evolutionary dynamics, population stratification, and comparative genomics in order to gain insights into the functional implications of observed variations with an emphasis on spike protein. 

## 2. Materials and Methods

### 2.1. State-Wise Distribution of SARS-CoV-2 Isolates Sequenced from INDIA

Complete genome sequences of 45,273 SARS-CoV-2 samples isolated from various States and UTs of India were retrieved from the GISAID database available as of 15 August 2021 (https://www.gisaid.org/; Last accessed 30 October 2021) [5]. Associated metadata were used for curation. Entries with PANGO lineage assignments, geographic location (States/UTs), and date information as dd-mm-yy/mm-yy were retained, leading to 42,989 entries (Dataset_1; Supplementary File S1). The entries were annotated with WHO nomenclature with respect to lineages, variants of interest (VoI), and variants of concern (VoC). 

Plots were generated to depict month-wise distribution of VoCs and VoIs for Indian States and UTs for Dataset_1 using packages ggplot 0.11.5 and plotnine 0.8.0 (https://pypi.org/project/ggplot/, https://pypi.org/project/plotnine/; Last accessed 30 October 2021) in Python (scripts submitted as Supplementary File S2). 

### 2.2. Evolutionary Dynamics of SARS-CoV-2 Isolates from India Using Complete Genomes 

High-quality complete genome sequences of 11,864 isolates of SARS-CoV-2 sampled from India (available as of 26 May 2021) were retrieved from the GISAID database (11,193 entries) and GenBank (671) which was termed Dataset_2 (Supplementary file S3). Lineages were assigned to these entries using NextClade (https://clades.nextstrain.org/; Last accessed 30 October 2021) [6]. PANGO lineages were assigned to 671 entries retrieved from the GenBank (https://www.ncbi.nlm.nih.gov/genbank/; Last accessed 30 October 2021) as these were missing in GenBank records. Dataset_2 with all lineage labels is provided (Supplementary File S3). A set of non-redundant genomes were identified using the standalone version of CD-HIT [22], resulting in 6,859 entries and termed as Dataset_3 (Supplementary File S4). Seven reference genome sequences identified by GISAID (EPI_ISL_466615, EPI_ISL_539548, EPI_ISL_418345, EPI_ISL_406862, EPI_ISL_412974, EPI_ISL_403932, and EPI_ISL_601443; Table 1) were added to Dataset_3. 

Multiple sequence alignment (MSA) of genomes was carried out using MAFFT v7.475 [23] with Wuhan (NC_045512.2) as the reference genome. The genome alignment was trimmed to remove UTRs using SEED 2 [24]. Recombination detection analysis was carried out using RDP5 [25] with a stringent p-value cutoff of 0.005 and positive prediction using at least 3 methods.

The nucleotide substitution model was selected using ModelTest based on the BIC criterion [26]. A maximum likelihood (ML)-based phylogenetic tree was built using a light version of IQ-TREE [27] in standalone mode. Molecular clock behavior was tested using TempEst [28]. A permutation test (with 10,000 permutations) was carried out using the ‘wPerm’ package (https://cran.r-project.org/web/packages/wPerm/index.html; Last accessed 30 October 2021) available in R (http://www.R-project.org/; Last accessed 30 October 2021) [29] to examine the significance of correlation coefficient between root-to-tip distance and time of isolation. The genome-wide nucleotide substitution rate (NSR) was estimated using BEAST v1.10.4 with a relaxed clock model and lognormal distribution [30]. GTR+I+gamma was used as a nucleotide substitution model. A constant size growth model was used as a demographic model. Markov Chain Monte Carlo (MCMC) simulations were run for 1 billion steps and sampled every 10,000 steps. Tracer v1.6 (http://tree.bio.ed.ac.uk/software/tracer/; Last accessed 30 October 2021) was used for assessing convergence and iTOL software (https://itol.embl.de/; Last accessed 30 October 2021) was used for the visualization of phylogenetic trees.

Parsimoniously informative (PI) sites from MSA (Dataset_3) were retrieved using MEGAX [31]. Linkage disequilibrium was calculated using the LIAN package [32] with 10,000 replicates. Population stratification was studied using the STRUCTURE program [33] and parallelSTRUCTURE package [34] capable of utilizing multi-core computing architecture. The previously described protocols were used for STRUCTURE runs [35,36]. Two sets of burn-in and burn lengths (150,000–300,000; 200,000–400,000) were used, and optimal clusters were chosen based on Evanno’s method [37] as implemented in the STRUCTURE HARVESTER [38] tool. 

The PI sites obtained for complete genomes, spike, and RdRp genes were subjected to principal component analysis (PCA) using the ADEgenet package v2.1.4 [39] (https://cran.r-project.org/web/packages/adegenet/index.html; Last accessed 30 October 2021) available in R4.1.1 software (https://cran.r-project.org/bin/windows/base/; Last accessed 30 October 2021). The PCA plots were generated using the software Minitab v17.1 (https://www.minitab.com/en-us/support/downloads/; Last accessed 30 October 2021). The MSA of complete genomes, spike and RdRp sequences were analyzed to estimate Shannon entropy using the tool SHIAT v1.1 [40]. 

### 2.3. Analyses of Spike gene and Protein Sequences of SARS-CoV-2 Isolates from India 

Nucleotide sequences belonging to the spike gene of Indian SARS-CoV-2 isolates were retrieved from Dataset_2 (Supplementary File S3) to analyze the diversification of spike across various lineages for a duration of 17 months (January 2020–May 2021) as well as to identify sites under selection in various States and UTs of India. The spike gene sequences containing ambiguous base(s) were removed, and the resultant dataset was designated as Dataset_4 (Supplementary File S5), which includes 9538 sequences. 

Sequences in Dataset_4 were delineated further based on the States/UTs from which these isolates were sampled. A set of non-redundant spike gene sequences were curated for every State and UT using the standalone version of CD-HIT, resulting in 3363 entries pan India. Separate MSA of the spike gene sequences was carried out for every State/UT using the MAFFT program available in SEED 2. Sites under positive selection in the spike gene (State/UT wise) were delineated with a p-value cutoff of 0.05 for SLAC, FEL [41], and MEME [42], whereas the cutoff was 0.9 for FUBAR [43] methods, which are available in the Datamonkey server [44]. 

The amino acid sequences corresponding to Dataset_4 were aligned to identify variable sites. The frequencies of substitutions at every position with reference to the Wuhan isolate were obtained. Substitutions listed for VoC and VoI as defined by the WHO were retrieved (source: https://www.cdc.gov/coronavirus/2019-ncov/variants/variant-info.html; accessed on 1 September 2021) and compared with sites under selection. 

The sites under selection were mapped on various domains such as the S1 N-terminal domain (NTD), receptor binding domain (RBD), SD1 and SD2 sub-domains, S1–S2 furin cleavage site, and S2 region as well as on a 3D structure of the spike protein (PDB ID: 7DF3; cryo-EM structure solved at resolution of 2.7Å) [45] using Biovia Discovery Studio software v17.1.0.16143 (https://www.3ds.com/products-services/biovia/products/; Last accessed 30 October 2021). The sites under selection that are part of experimentally validated B- and T-cell epitopes were curated as of 25 October 2021 from the IEDB database (https://www.iedb.org/; last accessed: 30 October 2021) [46] using a positive assay cutoff of 4 assays. 

## 3. Results

### 3.1. SARS-CoV-2 in India 

**Figure 1:**
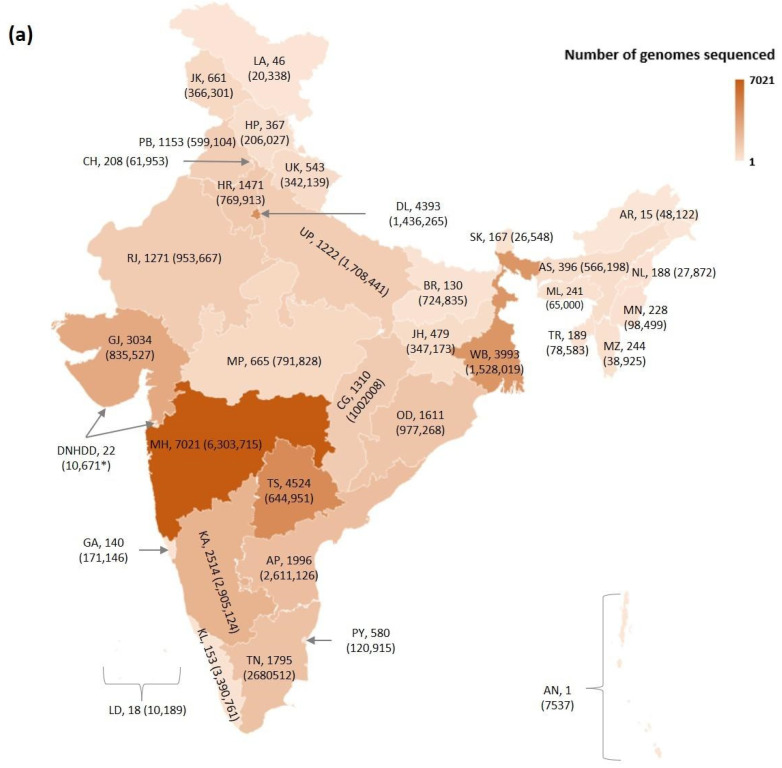

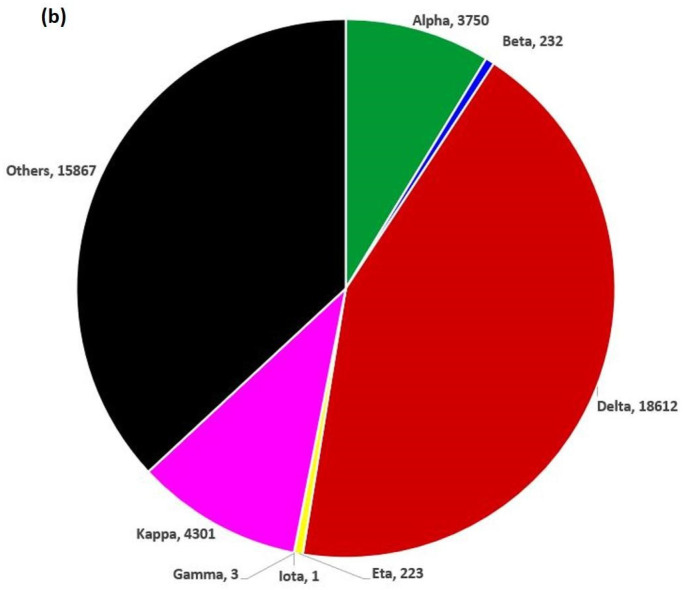
(**a**): Map of India depicting distribution of SARS-CoV-2 genomes sequenced from cases (shown in brackets) reported from the States and Union Territories (UTs) during January 2020 to July 2021. Two letter codes are used to label States and UTs. The number following the State/UT labels indicates the number of sequenced genomes. Note: States/UTs and their codes are as follows. Andaman and Nicobar Islands: AN; Andhra Pradesh: AP; Arunachal Pradesh: AR; Assam: AS; Bihar: BR; Chhattisgarh: CG; Chandigarh: CH; Dadra and Nagar Haveli and Daman and Diu: DNHDD; Delhi: DL; Goa: GA; Gujarat: GJ; Himachal Pradesh: HP; Haryana: HR; Jharkhand: JH; Jammu and Kashmir: JK; Karnataka: KA; Kerala: KL; Ladakh: LA; Lakshadweep: LD; Maharashtra: MH; Meghalaya: ML; Manipur: MN; Madhya Pradesh: MP; Mizoram: MZ; Nagaland: NL; Odisha: OD; Punjab: PB; Puducherry: PY; Rajasthan: RJ; Sikkim: SK; Tamil Nadu: TN; Tripura: TR; Telangana: TS; Uttarakhand: UK; Uttar Pradesh: UP; West Bengal: WB. Cases from DNHDD are as of 23 October 2021 and therefore marked with *. (**b**): Prevalence of WHO designated variants of concern (VoC) and variants of interest (VoI) based on pan-India genome sequencing coverage.

India contributed 45,277 genome sequences of SARS-CoV-2 isolates during January 2020 to July 2021, of which 42,989 sequences (95%) are included in our study (Dataset_1; Supplementary File S1) based on the availability of PANGO lineage information, month and year of isolation, and geographic location in terms of States/UTs. The distribution of genomes sequenced from the cases reported in various States/UTs of India is shown in Figure 1a. A lineage-wise prevalence of isolates sequenced at a pan-India scale is depicted in Figure 1b. 

As can be seen from Figure 1a, the highest sequencing coverage is seen for the states of Maharashtra followed by Telangana, West Bengal, and Gujarat, whereas among the UTs, Delhi has the highest sequencing coverage. The lineage-wise distribution of sequenced isolates from India indicates the presence of Alpha (9%), Beta (1%), Delta (43%), Eta (<0.1%), Gamma (<0.1%), Iota (<0.1%), and Kappa (10%) lineages (Figure 1b). The “Others” lineages (37%) include all isolates that do not belong to the WHO nomenclature of VoI and VoC that are sequenced in India. There are 290 distinct PANGO lineages, and their descendants are grouped as Others. These include lineage A and its descendants (≈1%), B and its descendants (98.9%; excluding Alpha, Beta, Delta, Eta, Iota, and Kappa), and descendants of C, L, P, and R (0.09%; excluding Gamma), indicating that B and its descendants dominate sequenced isolates from India, indicating their national prevalence (Dataset_1; Supplementary File S1).

**Figure 2:**
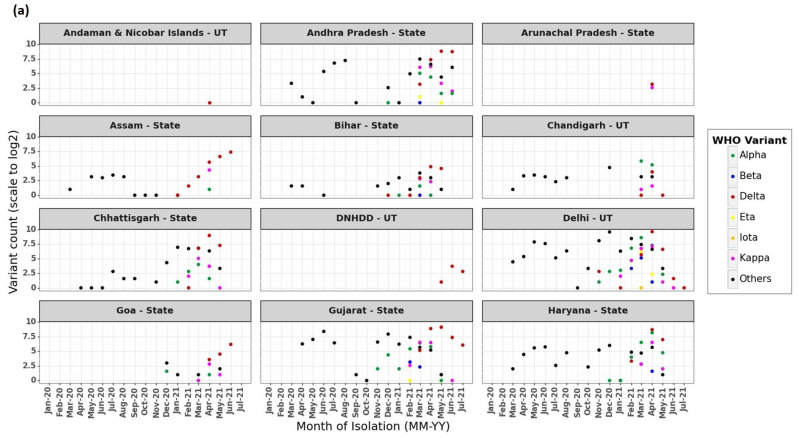

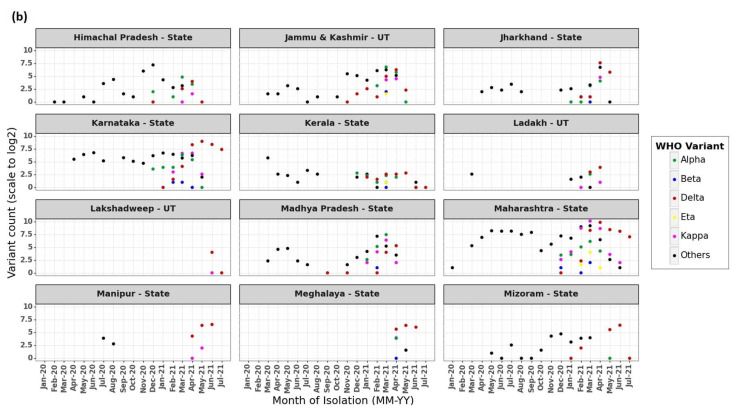

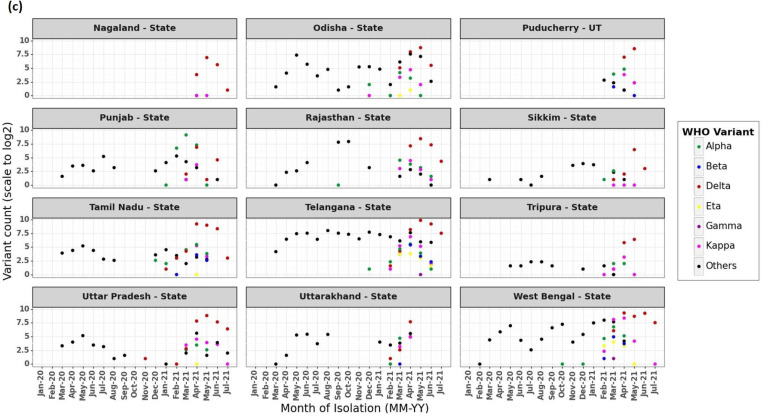
(**a**–**c**): Frequency distribution of VoC and VoI circulating in various States and UTs of India during 17 months (January 2020–July 2021) of the SARS-CoV-2 pandemic. Note: *X*-axis denotes month of isolation and *Y*-axis denotes variant count scaled to log 2.

The frequency distribution of isolates with respect to lineage data, geographic locations (States and UTs), and time of isolation is shown as Figure 2a–c. These plots indicate that the state of Maharashtra sequenced isolates since the onset of the pandemic (January 2020). The states West Bengal, Gujarat, Karnataka, Telangana, and Uttar Pradesh in addition to UTs of Delhi, Jammu, Kashmir, and Chandigarh have good sequencing coverage (starting from February–April 2020 until 15 August 2021) as compared to the rest of the country. Figure 2 also shows the presence of lineages Alpha, Beta, Delta, Eta, Iota, Gamma, and Kappa in addition to all Others (which includes lineages that are not designated as VoI and VoC) in most of the States/UTs during the pandemic with varying proportions. 

### 3.2. Evolutionary Dynamics of SARS-CoV-2 in India

Population genomics and phylogenomic studies require granular data of high resolution. Therefore, curated complete genome sequences of 11,864 isolates (Dataset_2; Supplementary File S3) were screened further to obtain a set of non-redundant genomes consisting of 6859 entries. The equivalence of WHO lineages with the clade definitions of NextClade and GISAID is provided for Dataset_3 (Supplementary File S4). Overall, the predominance of NextClade lineages 20A, 20B, and 21A was observed, which corresponds to GISAID clades G, GR, and GRY in the pan-India dataset. However, no data corresponding to WHO lineages Gamma and Iota (NextClade: 20J/501Y.V3 and 21F; GISAID: G, GR (Gamma), GH (Iota)) are part of our study, as they were eliminated during curation due to poor sequencing coverage and quality.

MSA of these sequences with reference genomes was carried out, and UTRs were trimmed. Recombination detection analysis confirmed a lack of recombination in Indian SARS-CoV-2 isolates. 

**Figure 3:**
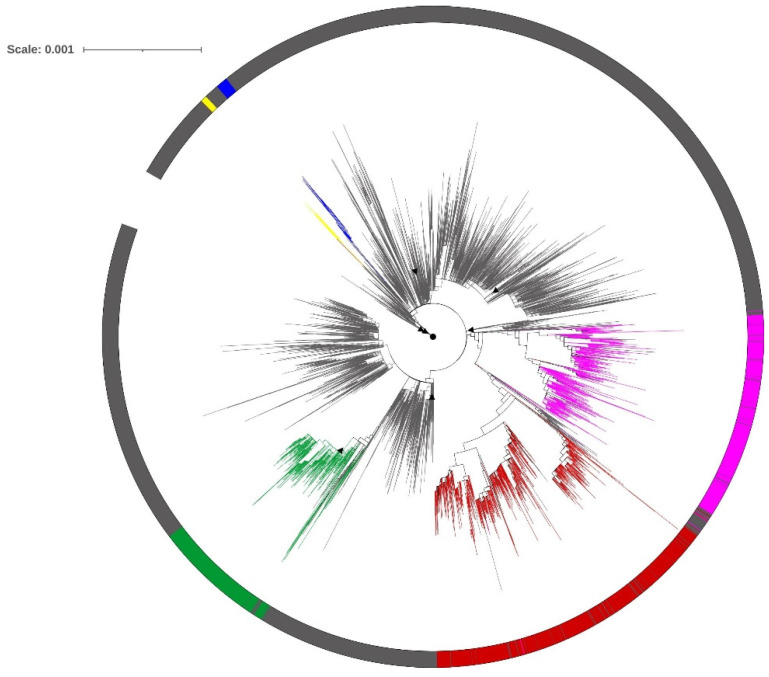
Phylogenetic tree derived using the Maximum Likelihood method for complete genome sequences (6859) of various SARS-CoV-2 lineages labeled as variants of concern and variants of interest. Lineages that are not designated as VoC/VoI are labeled as Others. Color codes: Alpha (green), Beta (blue), Delta (red), Kappa (magenta), Eta (yellow), and Others (gray). The Wuhan isolate (NC_045512.2) is denoted as a circle (black), and reference genomes are denoted with triangles (black).

The Maximum Likelihood (ML) tree generated using the GTR+I+gamma substitution model shows lineage-wise clustering of data (Figure 3) where Alpha (green), Delta (red), and Kappa (magenta) are seen as monophyletic taxa. Tree topologies are supported with >70% bootstrap values (not displayed). Others (gray) include all isolates that are not identified as VoI and VoC and are observed to have diversified into multiple branches. The members that are part of the diversified tree topologies of Others refer to the isolates of SARS-CoV-2 that emerged from several introductions of SARS-CoV-2 in India from various countries (spatial) and at different time points (temporal), which are sampled from multiple States/UTs. The presence of branches depicting the emergence of Beta (blue) and Eta (yellow) provide evidence for lineage diversification from the sub-groups that are designated as Others (gray) in this analysis. 

Root-to-tip regression analysis (Supplementary Figure S6) carried out for the complete genome-based phylogenetic tree revealed a high positive correlation (0.7) between root-to-tip distance and time of isolation. A permutation test rejected the null hypothesis of no correlation between root-to-tip distance and time of isolation with *p* < 0.001. The R^2^ for the fitted line of regression indicated the significance of the fit. 

Phylodynamic studies of Indian SARS-CoV-2 data using BEAST with a constant growth model were carried out to understand the evolution of the virus in India during January 2020 to May 2021. The genome-wide nucleotide substitution rate (NSR) computed for Indian SARS-CoV-2 data is estimated to be 6.73 × 10^−2^ subs/site/year (95% HPD 5 × 10^−3^, 7 × 10^−2^). The maximum clade credibility tree (not shown) substantiates the emergence of Delta and Kappa in India along with the circulation of various lineages. Among the VoCs, Alpha is the first to be sampled (2020.93; second week of December 2020) followed by Beta and Delta (2020.96; third week of December 2020), Eta and Kappa (2020.97; third week of December 2020), and Zeta (2020.98; fourth week of December 2020). Designation of the PANGO lineage P.2 as Zeta stands canceled as per the WHO guidelines as of 1 September 2021. 

**Figure 4:**
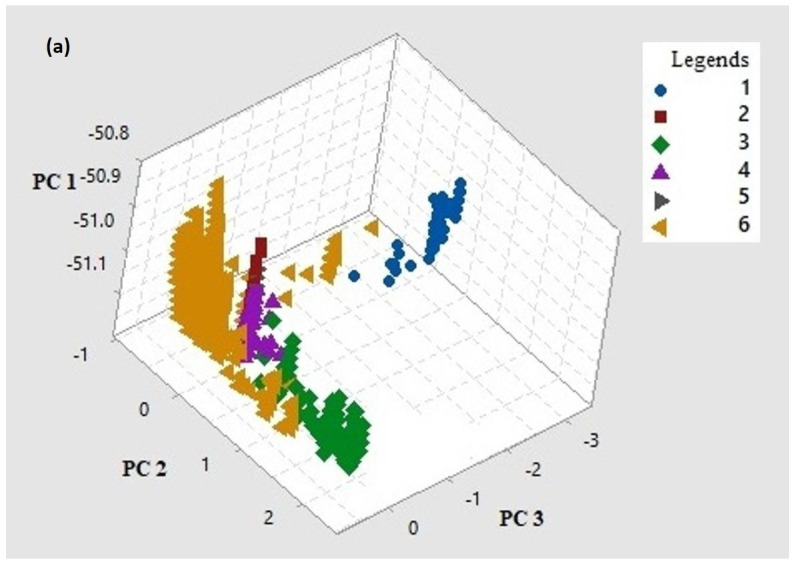

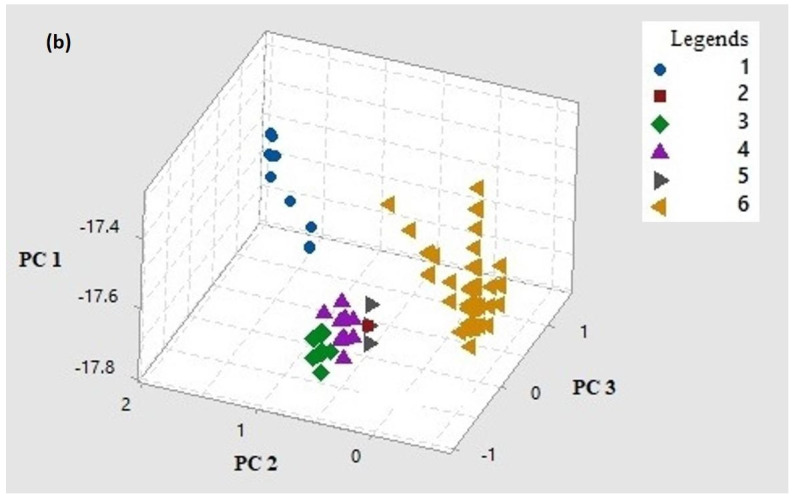

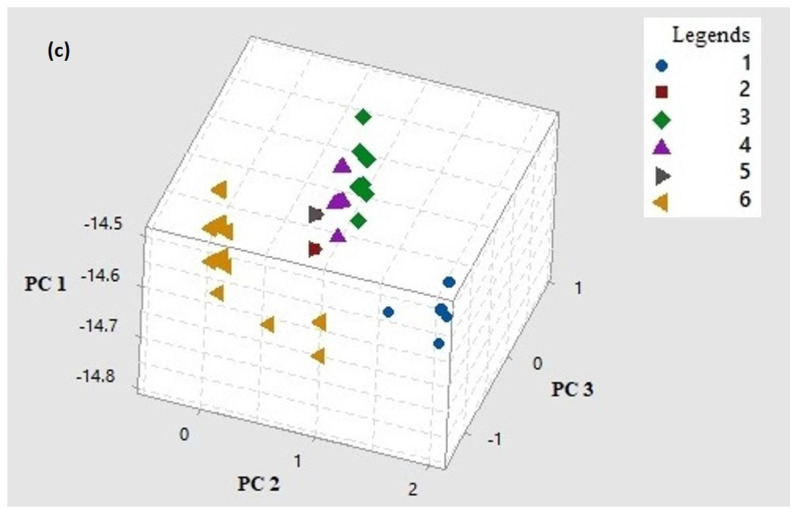
Cluster plots generated using Principal Component Analysis (PCA) of SARS-CoV-2: complete genome data (**a**); spike gene (**b**) and RdRp gene (**c**) for VoCs and VoIs designated as 1 (Alpha), 2 (Beta), 3 (Delta), 4 (Kappa), 5 (Eta), and 6 (Others).

MSA of complete genomes (Dataset_3) helped identify a total of 2618 parsimoniously informative (PI) sites across all ORFs of Indian isolates of SARS-CoV-2. PI sites were used to carry out PCA using whole genome data (Figure 4a) as well as for spike (Figure 4b) and RdRp (Figure 4c) genes. 

A genome-based PCA scatter plot generated for the first three PCs (accounting for 99.71% variance) revealed three major clusters, wherein Alpha (1) clustered independently. Delta (3) and Kappa (4) formed clusters next to the clusters formed by Beta (2), Eta (5), and Others (6). A few members of Alpha (1) and Others (6) are away from their respective clusters and therefore appear to form a continuum (Figure 4a). A spike-based PCA scatter plot (Figure 4b) for the first three PCs (accounting for 99.36% variance) shows three distinct clusters corresponding to Alpha (1), Others (6), and a cluster inclusive of Beta (2), Delta (3), Kappa (4), and Eta (5). An RdRp-based PCA scatter plot (Figure 4c) for the first three PCs (accounting for 99.25% variance) shows three clusters with Alpha (1), Others (6), and a cluster inclusive of Beta (2), Delta (3), Kappa (4), and Eta (5). However, points representing members of Beta (2), Delta (3), Kappa (4), and Eta (5) are distinct. A few members of Others (6) are away from its representative cluster and are equidistant from Others and Alpha clusters. The information content for complete genome, spike, and RdRp in terms of Summed Shannon Entropy Scores (SSES) are 62.43, 41.85, and 6.65, respectively. The ratio of SSES for genome to RdRp is 9.4 and spike to RdRp is 6.3.

**Figure 5:**
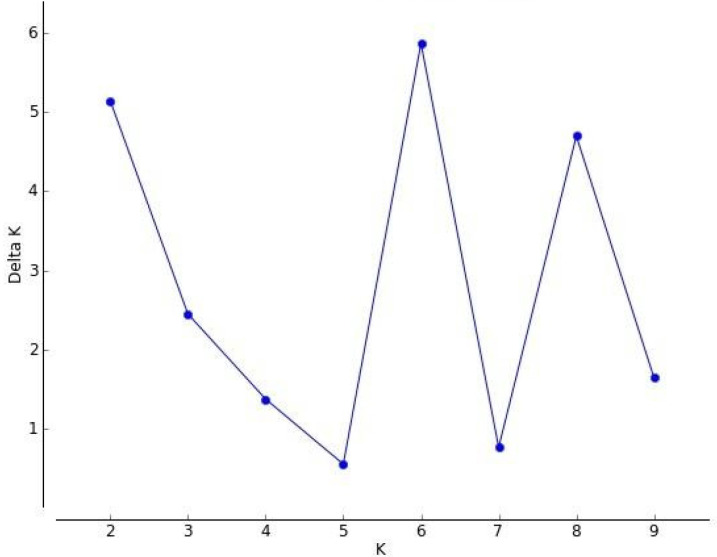
A plot of posterior probability for a given value of *K* vs. associated standard deviation (Δ*K*) to determine the optimal number of clusters representing the complete genome data of Indian SARS-CoV-2 isolates.

Genome-wide PI site analysis revealed low linkage disequilibrium (I^S^_A_ = 0.003), which makes the data amenable for population structure analyses using the STRUCTURE program. A major peak obtained at *K =* 6 indicated the presence of six clusters (C1–C6), each representing a subpopulation (Figure 5).

The presence of a second peak at 8 showed a further stratification of Kappa isolates. The isolates belonging to all the lineages were observed to have varying proportions of admixture, and based on the extent of admixture, these members were observed to cluster in C1–C6 (Supplementary File S7). The members belonging to individual clusters showed varying degrees of admixture with membership scores ranging from 0.93 (highest) and 0.05 (lowest) indicative of non-homogeneous clusters (C1–C6), with isolates belonging to more than one lineage. Clustering based on major membership scores (>0.8) indicates that 382 isolates (96%) of the Alpha lineage correspond to C1 along with 14 isolates (4%) of Others. Similarly, C2 comprises of 169 isolates (18%) of Kappa and 756 isolates (82%) of Others; C3 includes 714 isolates (99%) of Others and only 4 isolates (1%) of Kappa; C4 comprises of 40 isolates (5%) of Beta, 117 isolates (13%) of Delta, 20 isolates (2%) of Eta, 195 isolates (23%) of Kappa, and 491 isolates (57%) of Others; C5 comprises of 6 isolates (1%) of Kappa and 783 isolates (99%) of Others; the majority of C6 includes 744 isolates (93%) of Delta and 53 isolates (7%) of Others. Overall, the members of the Kappa lineage are observed to be distributed across C2–C5, whereas the majority of Delta lineage isolates are part of C6 with a few isolates in C4. An admixture was observed in all the clusters that include members of various lineages. Thus, three clusters identified with PCA analysis were further resolved into six clusters using both phylogeny and population stratification studies. 

### 3.3. Positive Selection and Mapping Mutations on 3D Structure of Spike Protein

As reported earlier, every State and UT had a prevalence of various lineages as the pandemic progressed (Figure 2a–c). Overall, 86 codon sites (of the total 1273 codons) were found to be under positive selection, leading to non-synonymous substitutions in Indian isolates belonging to various lineages. Of 86 sites, P681, E484, G142, and T95 are observed in at least 10 States/UTs and are common to Alpha, Delta, and Kappa variants sampled in India; of these, E484 is also sampled in Beta, Eta, and Gamma variants in India (Supplementary File S8). 

**Figure 6:**
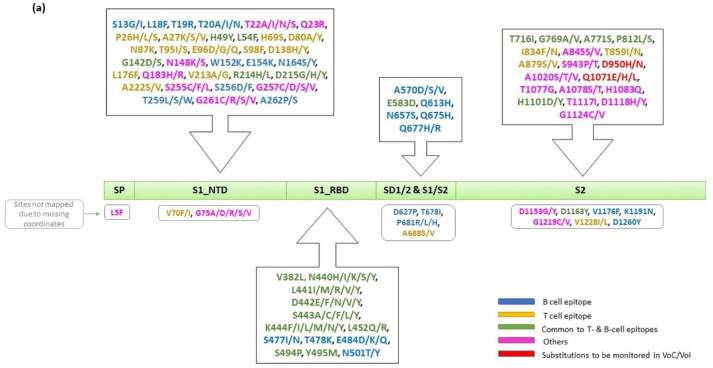

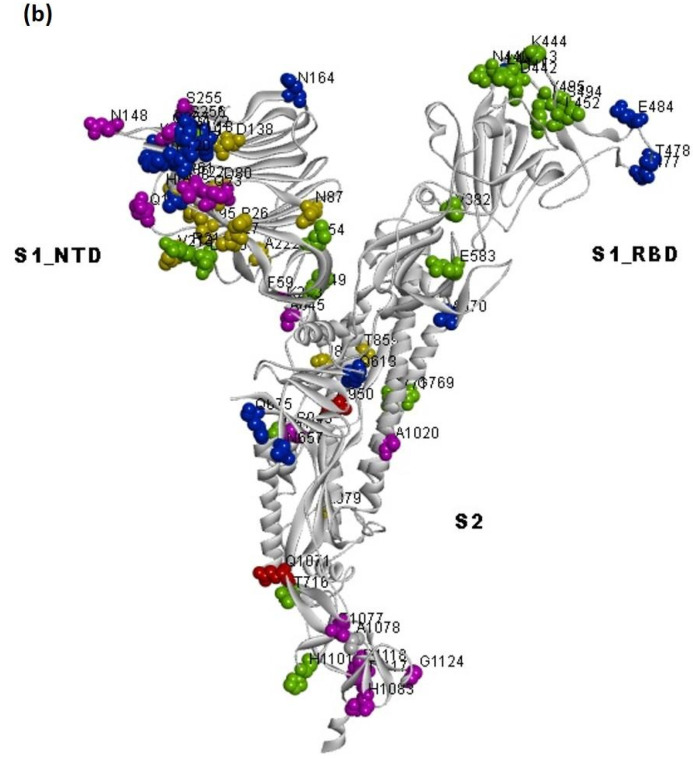
Sites under positive selection mapped on domain organization of spike (**a**,**b**) 3D structure (PDB ID: 7DF3). Note: Sites are color coded as B-cell epitopes (blue), T-cell epitopes (yellow), common to T- and B-cell epitopes (green), Others (magenta), and substitutions to be monitored in VoC/VoI (red).

The sites under selection were found to be distributed across the entire spike protein. They are part of signal peptide (#1), S1_N’ terminal domain (#36), S1_receptor-binding domain (#13), sub-domain 1/2 (SD1/SD2), S1/S2 cleavage region (#9), and S2 region (#27). Figure 6a,b show the mapping of sites under selection according to sequence and 3D structure (PDB ID: 7DF3) [45], respectively. Functional annotations of sites under selection are provided (Supplementary File S9). 

The accessible surface area (ASA) of spike trimer (PDB ID: 7DF3) was computed, and amino acids having ASA > 25% were termed accessible, which revealed that 48 residues (of 86 sites under selection) are present on the surface and 22 residues are partially buried (ASA < 25%). Of the 86 sites under selection, 38 and 45 mapped to experimentally validated T- and B-cell epitopes, respectively, and 20 sites are common to both T- and B-cell epitopes. Of these 20 sites, eight are partially buried. It is interesting to note that the substitutions sampled in sites under selection (N440H/I/K/S/Y, L441I/M/R/V/Y, D442E/F/N/V/Y, S443A/C/F/L/Y and K444F/I/L/M/N/Y) that belong to RBD are continuous, hypervariable, and are part of multiple experimentally validated conformational B-cell epitopes and T-cell epitopes (CD4) as per IEDB records. Two ACE-2 binding site residues E484Q/K/D and N501Y/T/S are found to be under selection. A total of 16 sites under selection could not be mapped onto 3D structures due to missing coordinates (Supplementary File S9). 

The sites P681, E484, G142, and T95 were under selection in 19, 13, 12, and 11 States/UTs, respectively. The selection site P681R/H/L is a part of the SD1/SD2 and S1/S2 cleavage domains where Pro is replaced by Arg (positively charged), His (positively charged upon protonation), and Leu (hydrophobic and aliphatic). P681R/H/L substitution is observed in 19 States/UTs and has been sampled in Alpha, Delta, and Kappa in India. This site is a part of an experimentally validated linear B-cell epitope. The site E484D/K/Q is part of S1_RBD and is a part of 31 B-cell conformational epitopes. The mutation E484K is acidic to basic substitution and alters the charge. This site, sampled in 13 States/UTs, is present in Alpha, Beta, Delta, Eta, Gamma, and Kappa variants observed in Indian isolates. The site G142D/S/- is a part of S1_NTD as well as that of three conformational B-cell epitopes and several T-cell (CD4 and CD8) epitopes, which was experimentally validated. Substitution of the flexible and hydrophobic Gly residue by a negatively charged Asp and hydrophilic Ser will impact the property profile as well as the local conformation of spike protein. This site is found in isolates sampled in Alpha, Delta, and Kappa variants from 12 States. The deletion at this site is observed in Kappa. The site T95I/S is a part of S1_NTD and a confirmed T-cell epitope. The substitution of Thr with Ile will impact hydrophilicity. It is prevalent in 11 States/UTs and is sampled in Alpha, Delta, and Kappa variants in India (Supplementary Files S8 and S9). 

In addition to the sites under positive selection in various States and UTs, MSA helped to identify 460 variable sites (totaling to 546 sites) in spike protein of Indian isolates. Of these, 17 variable sites that are functionally important and are sampled with significant frequency but are not under selection include S12F, K77T/M/R/N, R78M/S, F157(DEL)/S/L, Q218H/E, V341I, A344T, K417N/T, N439K/I/T, V445F/I/Y/G/C, G446V/A, Y453F, L455F, A520S, A701T/V/S, S982A, and V1230L/M (Supplementary File S10). 

These 17 significantly variable sites are part of signal peptide (#1), S1_N’ terminal domain (#4), S1_receptor-binding domain (#9), and S2 region (#3). No significantly variable site was found to be part of the SD1/SD2 and S1/S2 cleavage region. The computation of ASA revealed that of the 17 variable sites, seven are exposed on the surface (ASA > 25%), eight are partially buried, and the remaining two residues could not be mapped on the 3D structure (PDB ID: 7DF3) [45]. Of these, 13 and 11 residues are part of experimentally validated B- and T-cell epitopes, respectively, and eight are common to both types of epitopes. Sites K417, G446, Y453, and L455 are part of the ACE-2 binding site (Supplementary File S10). 

The N-linked glycosylation sites of spike protein that are under selection in Indian data include N657S (confirmed) and T20N (potential gain due to substitution), whereas the O-linked glycosylation site observed to be under selection is T678I/A. Four N-linked sites (N282, N616, N709, and N1074) are also observed to be variable (though with low frequency) in Indian isolates. The O-linked glycosylation sites (T323I/A and T678I/A) are seen to vary in Indian isolates (Supplementary Files S9 and S10). The sites under selection and significant variation reported in our study are a subset of mutations attributed to respective VoC and VoI (Table 2). 

## 4. Discussion

India ranks in second position with 15% of global prevalence in terms of total number of reported COVID-19 cases (based on WHO situation reports as on 30 August 2021; https://covid19.who.int; Last accessed 30 October 2021) and contributed ≈1.58% of complete genomes sequenced globally (based on GISAID statistics as on 30 August 2021). India has sequenced ≈0.14% of isolates from the confirmed cases through initiatives of various virology laboratories that are funded by the Central and State Governments. These independent sequencing efforts were followed by the Pan-India network, INSACOG initiative, funded by the Government of India (https://dbtindia.gov.in/insacog; Last accessed 30 October 2021). 

Sequencing coverage varies across India with representation from every State and UT (Figure 1a). The lineage-wise prevalence of isolates belonging to Alpha, Beta, Delta, Eta, Gamma, Iota, and Kappa, sequenced pan India, depicts how different lineages circulated during the pandemic and clearly indicates the emergence, decline, and sustained circulation of lineages in India (Figure 1b). In addition to VoCs and VoIs, the data of all other PANGO lineages that are in circulation in India are designated as Others in our study. As expected, Delta predominates all lineages due to its emergence in India, higher transmissibility, as well as the scaling up of sequencing efforts that coincided with the onset of the second wave. Previous study involving whole genome sequencing and analysis of ≈3000 whole genome sequences characterized from 20 states during January–September 2020 revealed the circulation of GR and GH clades (designated by GISAID) in India [9]. 

The mapping of high-resolution genomic data sequenced from various States and UTs revealed that although the extent of sequencing is highly variable, the presence of various SARS-CoV-2 lineages was observed during the pandemic at different time points (Figure 2a–c). This can be attributed to the importation/exportation of the SARS-CoV-2 isolates due to global/local travel, superspreading events, and limited awareness of COVID-appropriate behavior. Sequencing and analysis of ≈100 genomes of SARS-CoV-2 sampled across India reported multiple introductions from Europe, the USA, the Middle East, and Southeast and Central Asia, along with evidence for local transmission [3]. An independent study that analyzed data from the state of Gujarat for a period of six months pertaining to the first wave of the pandemic also reported more than 100 introductions of isolates to be responsible for the rapid spread of disease [47]. A report on the spread of VoCs (Alpha, Beta, and Gamma) and Kappa (VoI) in India along with their prevalence in various States as of March 2021 was documented recently [48]. 

The whole genome phylogenetic analysis reported in this study (Figure 3) further substantiates multiple introductions from other countries as well as the circulation of SARS-CoV-2 lineages across various States and UTs of India as evident from lineage-wise monophyletic clusters of VoCs, with the exception of a few isolates belonging to Alpha and Beta, which clustered with Others. The lineage designated as Others was observed to diversify into multiple tree topologies, which is in accordance with respective PANGO lineages, as expected. The phylogenetic clusters were independent of geographic proximity and time of isolation, which can be attributed to introductions due to global and interstate travel as well as urban to rural migration in India. 

Although recombination is responsible for the evolution and diversification of Beta coronaviruses, there are only early indications of potential recombination for SARS-CoV-2 [49]. Recent reports of recombination in isolates belonging to Alpha (B.1.1.7) from the United Kingdom have been observed [50]. In the present study, no significant evidence for recombination has been observed in the Indian isolates. However, improved sequencing coverage of co-infection cases might help to identify recombination events, if any, in the future. 

Regression analysis (root-to-tip distance) based on complete genomes of pan India isolates (Dataset_3; Supplementary File S4) depicted temporal signals. Similar observations have been reported earlier [51,52]. The NSR estimated using complete genomes of Indian isolates (6.73 × 10^−2^ subs/site/year; 95% HPD: 5 × 10^−3^ to 7 × 10^−2^) is higher as compared to that reported for Southeast Asia data (1.44 × 10^−3^ subs/site/year; 95% HPD 1.292 × 10^−3^ to 1.613 × 10^−3^) and global (representative) VoC isolates (6.5 × 10^−4^ subs/site/year; 95% HPD 0.58 − 0.77 × 10^−3^) [51,52,53]. The estimated NSR in Indian data is higher than the NSR of known RNA viruses and similar to that reported for viroids [54]. An increase in the sampling duration of Markov Chain Monte Carlo simulations might help to generate better NSR estimates; however, the size of the data and computation time required to estimate NSR poses challenges. Simulations were carried out with 1 billion steps that showed a mean rate convergence value of 500, which is in the required range >200. A higher value of NSR could also be attributed to the inclusion of samples over a period of 17 months (January 2020 to May 2021) that included the earliest isolate from Wuhan (12 January 2020; designated as Others) and the most recent isolate (7 May 2021; Delta). 

The estimated date of emergence of both Delta and Kappa is reported as October 2020. However, our estimates for the emergence of Delta and Kappa are in the third week of December 2020. An investigation pertaining to the deferred date of estimation in our analysis revealed that the earliest samples of Delta isolated from the state of Madhya Pradesh (Collection date: 7 September 2020) was not included in the present analysis, as it was submitted to GISAID on 9 June 2021 (ID: 2461258). The earliest entry of Delta in the curated dataset used for analysis is 12 December 2020, which corroborates with the estimated date of emergence of Delta in our study. Similarly, the first entry of Kappa in GISAID was recorded on 3 March 2020, for which genome data were deposited in April 2021. The latest entry of Kappa in the curated dataset used in this study is for 1 December 2020, which corroborates with the estimated date of emergence of Kappa in India as estimated in our study. The gap in actual vs. estimated dates of emergence of Delta and Kappa can be attributed to a lack of periodic surveillance, lag in sequencing, and delay in data submissions. 

The clustering patterns for VoIs and VoCs for spike and RdRp genes obtained by PCA were similar as compared to those derived from complete genomes. The SSES and their ratios explain the within and between variation associated with PCA clusters obtained for genome, spike, and RdRp. The outcome of exploratory PCA was further resolved using fine-level population stratification analysis into six clusters based on major membership scores. The isolates of Alpha clustered independently in both PCA and STRUCTURE analysis. In PCA plots, isolates of Delta clustered with Kappa (whole genome); with Kappa, Beta and Eta (Spike and RdRp), whereas the majority of Delta isolates formed an independent cluster in STRUCTURE analysis. The PCA outcome also indicates the relatedness of Delta and Kappa, as both diversified from the B.1.617 lineage. Isolates belonging to Kappa show membership to multiple clusters in STRUCTURE analysis, which is indicative of its overall higher variability and is also seen in the clustering pattern of Kappa isolates in the phylogenetic tree. All the clusters obtained using STRUCTURE included a small proportion of isolates with mixed ancestry (membership scores to more than one cluster). However, cluster C4 was characteristic with all Beta and Eta isolates, along with 12% of Delta and 28% of Kappa based on major membership scores. The clustering of multiple VoCs can be explained on the basis of the presence of a significant proportion of shared variable sites. The phylogenetic tree (Figure 3) also corroborates these observations wherein a subcluster of Delta is seen in proximity to a few Kappa isolates. These analyses together hint at adaptive variations, conferring distinct advantages in terms of increased transmissibility, reduced latency, and antigenic variation to SARS-CoV-2 isolates, some of which subsequently evolved to be designated as VoC and VoI.

The mapping of 86 sites under positive selection and 17 sites with significant variations in spike protein helped identify substitutions that are part of mutations specific to VoC and VoI (Table 2). However, a prior study reported positive selection in non-structural genes and absence of the same in spike based on an analysis of ≈3000 whole genome sequences characterized from 20 states sampled during January–September 2020 [9]. Subsequently, an independent study reported multiple sites under positive selection in the spike gene using representative data [55], and 10 sites reported in their study overlap with the outcome of this study. 

The substitutions T20N, A222V, and Q677H/R are under selection in Indian isolates that are associated with either N- or O-linked glycosylation. The spike protein has three O-linked and 22 N-linked experimentally validated glycosylation sites, of which N343 is conserved [56,57]. Variations have been observed in the Indian and global data at these sites, which hints at differential glycosylations of the spike protein. Additional studies are required to validate the glycosylation of the spike protein and its implications in the context of antigenic variation in general and epitope masking in particular. A222V is the second most frequent amino acid substitution in global data next to D614G [58]. A222V is a significant substitution associated with the Delta variant and is also sampled with high frequency in Kappa isolates in India. The site Q677H/R is a convergent substitution that is sampled in Indian and global Delta, Eta, and Kappa variants. This site is close to the polybasic furin cleavage site [59]. 

Sites under selection are distributed over the entire spike protein, the S1 region containing NTD and RBD, which was observed to harbor a maximum number of sites under selection (Figure 6a,b) and significantly variable sites. These two domains are functionally important not only for maintaining a trimer conformation of spike and mediating host–receptor interactions but also as a predominant antigenic region with evidence for several monoclonal and polyclonal antibody binding sites, neutralizing sites as well as CD4 and CD8-responsive regions [60,61]. The variable sites N439K and Y453F, L455F and E484K are known to escape neutralization by therapeutic antibodies REGN10987 and REGN10933, respectively [62,63,64]. The mutations E484K/Q, L452R, and S494P escape neutralization by LY-CoV555 antibodies, whereas K417N/T are escape mutants of LY-CoV016 [18,65,66,67]. It is important to note that L452R, E484K/Q (ACE-2 binding site), and S494P are under selection in Indian isolates (Supplementary File S9). Additionally, the reduced neutralizing antibody response for other monoclonal antibodies and escape from cellular immune response in case of HLA-24 for L452R substitution has been reported [16,68,69]. Previous studies have reported reduced binding or escape from neutralization from four clinically approved therapeutic monoclonal antibodies, specifically for isolates of the Delta lineage. The substitutions in the Delta lineage were found to be responsible for the complete loss of neutralization by NTD-specific monoclonal antibodies and partial and/or complete loss by the RBD- and non-RBD-specific monoclonal antibodies [18,67]. The site under selection T478K reported in this study, attributed to the Delta lineage, is a major contributor to the neutralization site of multiple monoclonal antibodies [70]. V341 and A344 are antibody-binding sites for the VIR-7831 antibody, and we observed V341I and A344T substitutions. Similarly, A344 and K444 are binding residues of the antibody VIR-7832 [62], and Indian isolates are found to have A344T and K444N/R/M/I/F/L/Y substitutions that might negatively impact antibody binding affinity, which needs to be investigated. V341, A344, and K444 are neutralizing antibody-binding sites for antibody S309 [71], and the substitutions observed in Indian isolates include V341I, A344T, and K444N/R/M/I/F/L/Y. Similarly, N439 is critical for binding to antibody VHH-72 [62], and N439K substitution was sampled in Indian isolates. The variable sites N439K/I/T, V445F/I/Y/G/C, and G446V/A are flanking a segment under selection consisting of N440H/I/K/S/Y, L441I/M/R/V/Y, D442E/F/N/V/Y, S443A/C/F/L/Y, and K444F/I/L/M/N/Y, and therefore, the mutations in the region 439–446 need to be monitored, as it is a major antigenic site that is part of the binding sites of several neutralizing antibodies as well as experimentally validated T-cell epitopes, conferring both humoral and cellular immunity [72] (Supplementary Files S9 and S10). A significantly large number of antibodies recognize the regions at the periphery of the ACE-2 binding site, and therefore, the substitutions at those sites in VoCs impact the binding of antibodies without any impact on ACE-2 binding efficiency [67].

The impact of naturally occurring substitutions on the vaccine-mediated neutralization of VoIs and VoCs has been systematically studied using pseudo virus constructs [73,74]. The substitutions L452R (sampled in Delta and Kappa), E484K (sampled in Alpha, Beta, Gamma, and Eta), and N501Y (sampled in Alpha, Beta, Gamma, and Delta), which were observed to be under selection in Indian SARS-CoV-2 isolates, are reported to impact vaccine-mediated neutralization titers when tested for mRNA-1273 (Moderna) and BNT162b2 (Pfizer) vaccines [73,74,75,76,77,78]. E484Q (present in Kappa) has been observed to reduce neutralization when tested against BNT162b2 (Pfizer), but its impact is relatively lower as compared to E484K [78]. The substitutions L452R and N501Y have been predicted to impact the neutralization of ChAdOx1 (Covishield) and BBV152 (Covaxin) vaccines [79]. The significantly variable site K417N independently does not impact the efficacy of the vaccines mRNA-1273 (Moderna), BNT162b2 (Pfizer), and Sputnik V, but the same mutation in combination with E484K and N501Y (observed in Beta) is reported to provide an immune escape advantage when tested for mRNA-1273 (Moderna), BNT162b2 (Pfizer), and Sputnik V vaccines [73,74,75,76,77,80]. However, the substitution K417T in combination with E484K and N501Y (present in Gamma) showed limited reduction in neutralization as compared to the Beta variants when tested against the BNT162b2 vaccine [74,77]. The vaccines ChAdOx1 (Covishield), BBV152 (Covaxin), and Sputnik V are licensed in India.

In conclusion, this study provides an account of pan-India diversity of SARS-CoV-2 using complete genome data, which were sequenced for the purpose of pandemic surveillance and deposited in public domain databases. Although the sequencing coverage varied significantly across India, genomic data from every State and UT have been generated. Isolates belonging to all known VoCs and VoIs such as Alpha, Beta, Delta, Eta, Gamma, Iota, and Kappa were in circulation at different time points. Phylogenetic analysis and evolutionary dynamics depicted a lack of spatio-temporal clustering of isolates as expected during the pandemic. Indian isolates of SARS-CoV-2 are observed to harbor mutations in various genomic regions, and the substitutions in the spike protein in particular explain the potential impact on various functions including antigenic diversity and immune escape.

## Figures and Tables

**Table 1:** Details of the SARS-CoV-2 reference sequences (global) used for phylogenetic analysis. Note: * Exact date of isolation is not available for this entry.

Accession ID	GISAID Clade	Pango Lineage	Country of Isolation	Date of Isolation
EPI_ISL_466615	GR	B.1.1.1	England	26 June 2020
EPI_ISL_539548	GV	B.1.177	Spain	26 June 2020
EPI_ISL_418345	GH	B.1	Canada	February 2020 *
EPI_ISL_406862	G	B.1	Germany	28 January 2020
NC_045512.2	L	B	Wuhan, China	30 December 2019
EPI_ISL_412974	V	B.2	Italy	29 January 2020
EPI_ISL_403932	S	A	Guangdong, China	14 January 2020
EPI_ISL_601443	GRY	B.1.1.7	England	20 September 2020

**Table 2:** List of sites in spike protein that are under positive selection or significantly variable, which map to substitutions sampled in VoC/VoI. Highly variable sites are shown as grey filled rows.

Codon	Observed Substitutions	Substitution Attributed to VoC/VoI
19	I, R	T19R (Delta)
20	A, I, N	T20N (Gamma)
26	H, L, S	P26S (Gamma)
69	S, Y, -	H69- (Alpha, Eta)
80	A, G, H, N, Y	D80A (Beta)
95	I, S	T95I (Delta, Kappa)
138	H, Y	D138Y (Gamma)
142	D, S, -	G142D (Delta, Kappa)
154	K	E154K (Kappa)
157	S, L, -	F157- (Delta), F157S (Alpha)
215	G, H, Y	D215G (Beta)
222	S, V	A222V (Delta)
417	N, T	K417N (Beta), K417T (Gamma)
452	M, Q, R	L452R (Delta, Kappa)
478	I, K	T478K (Delta)
484	D, K, Q	E484K (Alpha)
501	S, T, Y	N501Y (Alpha, Beta, Gamma)
570	D, S, V	A570D (Alpha)
681	H, L, R	P681H (Alpha), P681R (Delta and Kappa)
701	T, V, S	A701V (Beta)
716	I	T716I (Alpha)
950	H, N	D950N (Delta)
982	A	S982A (Alpha)
1071	E, H, L	Q1071H (Kappa)
1118	H, Y	D1118H (Alpha)
1191	N	K1191N (Alpha)

## Data Availability

Primary data have been obtained from public domain resources with appropriate citation and listed in supplementary files. The derived data, as applicable, have been provided as supplementary files.

## Supplementary Materials

The following are available online at https://www.mdpi.com/article/10.3390/v13112238/s1, Supplementary file S1: List of GISAID IDs with metadata of SARS-CoV-2 sequences of isolates sampled from various States/Union Territories of India (Dataset_1: 42,989 entries; as of 15 August 2021), Supplementary file S2: Python script for generating States/UT wise plots of WHO designated VoC and VoI., Supplementary file S3: Curated GISAID and GenBank IDs and associated metadata of SARS-CoV-2 genome sequences from India (Dataset_2: 11,864 entries; as of 26 May 2021), Supplementary file S4: List of GISAID and GenBank IDs for non-redundant SARS-CoV-2 genomes from India used for evolutionary analysis (Dataset_3: 6859 entries), Supplementary file S5: List of GISAID and GenBank IDs corresponding to Spike gene data (Dataset_5: 9538), Supplementary file S6: Regression plot for Root-to-tip distances vs time of isolation of SARS-CoV-2 isolates from India., Supplementary file S7: Bar plot of fine level population structure of SARS-CoV-2 isolates from India., Supplementary file S8: Sites under positive selection in spike gene sequences belonging to SARS-CoV-2 isolates from India, Supplementary file S9: Functional annotations for Sites under positive selection in spike gene sequences belonging to SARS-CoV-2 isolates from India, Supplementary file S10: Functional annotations of significantly variable sites in spike protein sequences belonging to SARS-CoV-2 isolates from India.

## References

[1] Andrews M., Areekal B., Rajesh K., Krishnan J., Suryakala R., Krishnan B., Muraly C., Santhosh P. (2020). First confirmed case of COVID-19 infection in India: A case report. Indian J. Med. Res..

[2] Yadav P.D., Potdar V.A., Choudhary M.L., Nyayanit D.A., Agrawal M., Jadhav S.M., Majumdar T.D., Shete-Aich A., Basu A., Abraham P. (2020). Full-genome sequences of the first two SARS-CoV-2 viruses from India. Indian J. Med. Res..

[3] Kumar P., Pandey R., Sharma P., Dhar M.S. (2020). Integrated genomic view of SARS-CoV-2 in India. Wellcome Open Res..

[4] Gupta V., Bhoyar R.C., Jain A., Srivastava S., Upadhayay R., Imran M., Jolly B., Divakar M.K., Sharma D., Sehgal P. (2020). Asymptomatic reinfection in 2 healthcare workers from India with genetically distinct severe acute respiratory syndrome Coronavirus 2. Clin. Infect. Dis..

[5] Shu Y., McCauley J. (2017). GISAID: Global initiative on sharing all influenza data–from vision to reality. Eurosurveillance.

[6] Hadfield J., Megill C., Bell S.M., Huddleston J., Potter B., Callender C., Sagulenko P., Bedford T., Neher R.A. (2018). Nextstrain: Real-time tracking of pathogen evolution. Bioinformatics.

[7] Rambaut A., Holmes E.C., O’Toole Á., Hill V., McCrone J.T., Ruis C., du Plessis L., Pybus O.G. (2020). A dynamic nomenclature proposal for SARS-CoV-2 lineages to assist genomic epidemiology. Nat. Microbiol..

[8] Yadav P.D., Nyayanit D.A., Majumdar T., Patil S., Kaur H., Gupta N., Shete A.M., Pandit P., Kumar A., Aggarwal N. (2021). An Epidemiological Analysis of SARS-CoV-2 Genomic Sequences from Different Regions of India. Viruses.

[9] Potdar V., Vipat V., Ramdasi A., Jadhav S., Pawar-Patil J., Walimbe A., Patil S.S., Choudhury M.L., Shastri J., Agrawal S. (2021). Phylogenetic classification of the whole-genome sequences of SARS-CoV-2 from India & evolutionary trends. Indian J. Med. Res..

[10] Gupta A., Sabarinathan R., Bala P., Donipadi V., Vashisht D., Katika M.R., Kandakatla M., Mitra D., Dalal A., Bashyam M.D. (2021). A comprehensive profile of genomic variations in the SARS-CoV-2 isolates from the state of Telangana, India. J. Gen. Virol..

[11] Radhakrishnan C., Divakar M.K., Jain A., Viswanathan P., Bhoyar R.C., Jolly B., Imran M., Sharma D., Rophina M., Ranjan G. (2021). Initial insights into the genetic epidemiology of SARS-CoV-2 isolates from Kerala suggest local spread from limited introductions. Front. Genet..

[12] Dhar M.S., Marwal R., Radhakrishnan V.S., Ponnusamy K., Jolly B., Bhoyar R.C., Sardana V., Naushin S., Rophina M., Mellan T.A. (2021). Genomic characterization and epidemiology of an emerging SARS-CoV-2 variant in Delhi, India. Science.

[13] Pattabiraman C., Habib F., Harsha P.K., Rasheed R., Prasad P., Reddy V., Dinesh P., Damodar T., Hosallimath K., George A.K. (2020). Genomic epidemiology reveals multiple introductions and spread of SARS-CoV-2 in the Indian state of Karnataka. PLoS ONE.

[14] Kumar N., Hameed S.K.S., Babu G.R., Venkataswamy M.M., Dinesh P., Bg P.K., John D.A., Desai A., Ravi V. (2021). Descriptive epidemiology of SARS-CoV-2 infection in Karnataka state, South India: Transmission dynamics of symptomatic vs. asymptomatic infections. EClinicalMedicine.

[15] Joshi M., Puvar A., Kumar D., Ansari A., Pandya M., Raval J., Patel Z., Trivedi P., Gandhi M., Pandya L. (2021). Genomic variations in SARS-CoV-2 genomes from Gujarat: Underlying role of variants in disease epidemiology. Front. Genet..

[16] Cherian S., Potdar V., Jadhav S., Yadav P., Gupta N., Das M., Rakshit P., Singh S., Abraham P., Panda S. (2021). SARS-CoV-2 Spike Mutations, L452R, T478K, E484Q and P681R, in the Second Wave of COVID-19 in Maharashtra, India. Microorganisms.

[17] Shastri J., Parikh S., Agrawal S., Chatterjee N., Pathak M., Chaudhary S., Sharma C., Kanakan A., Srinivasa Vasudevan J., Maurya R. (2021). Clinical, Serological, Whole Genome Sequence Analyses to Confirm SARS-CoV-2 Reinfection in Patients from Mumbai, India. Front. Med..

[18] Mlcochova P., Kemp S., Dhar M.S., Papa G., Meng B., Ferreira I.A., Datir R., Collier D.A., Albecka A., Singh S. (2021). SARS-CoV-2 B. 1.617. 2 Delta variant replication and immune evasion. Nature.

[19] Sherry S.T., Karsch-Mizrachi I., Sayers E.W., Cavanaugh M., Clark K., Pruitt K.D., Schoch C.L. (2020). GenBank. Nucleic Acids Res..

[20] Rani P.R., Imran M., Lakshmi J.V., Jolly B., Jain A., Surekha A., Senthivel V., Chandrasekhar P., Srinivasulu D., Bhoyar R.C. (2021). Symptomatic reinfection of SARS-CoV-2 with spike protein variant N440K associated with immune escape. J. Med. Virol..

[21] Sarkar R., Mitra S., Chandra P., Saha P., Banerjee A., Dutta S., Chawla-Sarkar M. (2021). Comprehensive analysis of genomic diversity of SARS-CoV-2 in different geographic regions of India: An endeavour to classify Indian SARS-CoV-2 strains on the basis of co-existing mutations. Arch. Virol..

[22] Li W., Godzik A. (2006). Cd-hit: A fast program for clustering and comparing large sets of protein or nucleotide sequences. Bioinformatics.

[23] Katoh K., Rozewicki J., Yamada K.D. (2019). MAFFT online service: Multiple sequence alignment, interactive sequence choice and visualization. Brief. Bioinform..

[24] Větrovský T., Baldrian P., Morais D. (2018). SEED 2: A user-friendly platform for amplicon high-throughput sequencing data analyses. Bioinformatics.

[25] Martin D.P., Varsani A., Roumagnac P., Botha G., Maslamoney S., Schwab T., Kelz Z., Kumar V., Murrell B. (2021). RDP5: A computer program for analyzing recombination in, and removing signals of recombination from, nucleotide sequence datasets. Virus Evol..

[26] Whelan S., Allen J.E., Blackburne B.P., Talavera D. (2015). ModelOMatic: Fast and automated model selection between RY, nucleotide, amino acid, and codon substitution models. Syst. Biol..

[27] Trifinopoulos J., Nguyen L.-T., von Haeseler A., Minh B.Q. (2016). W-IQ-TREE: A fast online phylogenetic tool for maximum likelihood analysis. Nucleic Acids Res..

[28] Rambaut A., Lam T.T., Max Carvalho L., Pybus O.G. (2016). Exploring the temporal structure of heterochronous sequences using TempEst (formerly Path-O-Gen). Virus Evol..

[29] Team R.C. (2013). R: A Language and Environment for Statistical Computing. http://www.R-project.org/.

[30] Drummond A.J., Ho S.Y.W., Phillips M.J., Rambaut A. (2006). Relaxed phylogenetics and dating with confidence. PLoS Biol..

[31] Kumar S., Stecher G., Li M., Knyaz C., Tamura K. (2018). MEGA X: Molecular evolutionary genetics analysis across computing platforms. Mol. Biol. Evol..

[32] Haubold B., Hudson R.R. (2000). LIAN 3.0: Detecting linkage disequilibrium in multilocus data. Bioinformatics.

[33] Pritchard J.K., Stephens M., Donnelly P. (2000). Inference of population structure using multilocus genotype data. Genetics.

[34] Besnier F., Glover K.A. (2013). ParallelStructure: AR package to distribute parallel runs of the population genetics program STRUCTURE on multi-core computers. PLoS ONE.

[35] Waman V.P., Kolekar P.S., Kale M.M., Kulkarni-Kale U. (2014). Population structure and evolution of Rhinoviruses. PLoS ONE.

[36] Kasibhatla S.M., Kinikar M., Limaye S., Kale M.M., Kulkarni-Kale U. (2020). Understanding evolution of SARS-CoV-2: A perspective from analysis of genetic diversity of RdRp gene. J. Med. Virol..

[37] Evanno G., Regnaut S., Goudet J. (2005). Detecting the number of clusters of individuals using the software STRUCTURE: A simulation study. Mol. Ecol..

[38] Earl D.A. (2012). STRUCTURE HARVESTER: A website and program for visualizing STRUCTURE output and implementing the Evanno method. Conserv. Genet. Resour..

[39] Jombart T. (2008). adegenet: A R package for the multivariate analysis of genetic markers. Bioinformatics.

[40] Humphreys I., Fleming V., Fabris P., Parker J., Schulenberg B., Brown A., Demetriou C., Gaudieri S., Pfafferott K., Lucas M. (2009). Full-length characterization of hepatitis C virus subtype 3a reveals novel hypervariable regions under positive selection during acute infection. J. Virol..

[41] Pond S.L.K., Frost S.D. (2005). Datamonkey: Rapid detection of selective pressure on individual sites of codon alignments. Bioinformatics.

[42] Murrell B., Wertheim J.O., Moola S., Weighill T., Scheffler K., Kosakovsky Pond S.L. (2012). Detecting individual sites subject to episodic diversifying selection. PLoS Genet..

[43] Murrell B., Moola S., Mabona A., Weighill T., Sheward D., Kosakovsky Pond S.L., Scheffler K. (2013). FUBAR: A fast, unconstrained bayesian approximation for inferring selection. Mol. Biol. Evol..

[44] Weaver S., Shank S.D., Spielman S.J., Li M., Muse S.V., Kosakovsky Pond S.L. (2018). Datamonkey 2.0: A modern web application for characterizing selective and other evolutionary processes. Mol. Biol. Evol..

[45] Xu C., Wang Y., Liu C., Zhang C., Han W., Hong X., Wang Y., Hong Q., Wang S., Zhao Q. (2021). Conformational dynamics of SARS-CoV-2 trimeric spike glycoprotein in complex with receptor ACE2 revealed by cryo-EM. Sci. Adv..

[46] Vita R., Mahajan S., Overton J.A., Dhanda S.K., Martini S., Cantrell J.R., Wheeler D.K., Sette A., Peters B. (2019). The immune epitope database (IEDB): 2018 update. Nucleic Acids Res..

[47] Raghwani J., du Plessis L., McCrone J.T., Hill S.C., Parag K.V., Thézé J., Kumar D., Puvar A., Pandit R., Pybus O.G. (2021). Genomic epidemiology of early SARS-CoV-2 transmission dynamics in Gujarat, India. medRxiv.

[48] Singh J., Rahman S.A., Ehtesham N.Z., Hira S., Hasnain S.E. (2021). SARS-CoV-2 variants of concern are emerging in India. Nat. Med..

[49] González-Candelas F., Shaw M.-A., Phan T., Kulkarni-Kale U., Paraskevis D., Luciani F., Kimura H., Sironi M. (2021). One year into the pandemic: Short-term evolution of SARS-CoV-2 and emergence of new lineages. Infect. Genet. Evol..

[50] Jackson B., Boni M.F., Bull M.J., Colleran A., Colquhoun R.M., Darby A.C., Haldenby S., Hill V., Lucaci A., McCrone J.T. (2021). Generation and transmission of interlineage recombinants in the SARS-CoV-2 pandemic. Cell.

[51] Zhu M., Shen J., Zeng Q., Tan J.W., Kleepbua J., Chew I., Law J.X., Chew S.P., Tangathajinda A., Latthitham N. (2021). Molecular phylogenesis and spatiotemporal spread of SARS-CoV-2 in Southeast Asia. Front. Public Health.

[52] Kistler K., Huddleston J., Bedford T. (2021). Rapid and parallel adaptive mutations in spike S1 drive clade success in SARS-CoV-2. bioRxiv.

[53] Tay J.H., Porter A.F., Wirth W., Duchene S. (2021). The emergence of SARS-CoV-2 variants of concern is driven by acceleration of the evolutionary rate. medRxiv.

[54] Holmes E.C. (2010). The comparative genomics of viral emergence. Proc. Natl. Acad. Sci. USA.

[55] López-Cortés G.I., Palacios-Pérez M., Zamudio G.S., Veledíaz H.F., Ortega E., José M.V. (2021). Neutral evolution test of the spike protein of SARS-CoV-2 and its implications in the binding to ACE2. Sci. Rep..

[56] Pokhrel S., Kraemer B.R., Burkholz S., Mochly-Rosen D. (2021). Natural variants in SARS-CoV-2 Spike protein pinpoint structural and functional hotspots with implications for prophylaxis and therapeutic strategies. Sci. Rep..

[57] Watanabe Y., Allen J.D., Wrapp D., McLellan J.S., Crispin M. (2020). Site-specific glycan analysis of the SARS-CoV-2 spike. Science.

[58] Hodcroft E.B., Zuber M., Nadeau S., Vaughan T.G., Crawford K.H., Althaus C.L., Reichmuth M.L., Bowen J.E., Walls A.C., Corti D. (2021). Spread of a SARS-CoV-2 variant through Europe in the summer of 2020. Nature.

[59] Hodcroft E.B., Domman D.B., Snyder D.J., Oguntuyo K., Van Diest M., Densmore K.H., Schwalm K.C., Femling J., Carroll J.L., Scott R.S. (2021). Emergence in late 2020 of multiple lineages of SARS-CoV-2 Spike protein variants affecting amino acid position 677. MedRxiv.

[60] Dan J.M., Mateus J., Kato Y., Hastie K.M., Yu E.D., Faliti C.E., Grifoni A., Ramirez S.I., Haupt S., Frazier A. (2021). Immunological memory to SARS-CoV-2 assessed for up to 8 months after infection. Science.

[61] Hastie K.M., Li H., Bedinger D., Schendel S.L., Dennison S.M., Li K., Rayaprolu V., Yu X., Mann C., Zandonatti M. (2021). Defining variant-resistant epitopes targeted by SARS-CoV-2 antibodies: A global consortium study. Science.

[62] Thomson E.C., Rosen L.E., Shepherd J.G., Spreafico R., da Silva Filipe A., Wojcechowskyj J.A., Davis C., Piccoli L., Pascall D.J., Dillen J. (2021). Circulating SARS-CoV-2 spike N439K variants maintain fitness while evading antibody-mediated immunity. Cell.

[63] Baum A., Fulton B.O., Wloga E., Copin R., Pascal K.E., Russo V., Giordano S., Lanza K., Negron N., Ni M. (2020). Antibody cocktail to SARS-CoV-2 spike protein prevents rapid mutational escape seen with individual antibodies. Science.

[64] Weisblum Y., Schmidt F., Zhang F., DaSilva J., Poston D., Lorenzi J.C., Muecksch F., Rutkowska M., Hoffmann H.-H., Michailidis E. (2020). Escape from neutralizing antibodies by SARS-CoV-2 spike protein variants. Elife.

[65] Liu Z., VanBlargan L.A., Bloyet L.-M., Rothlauf P.W., Chen R.E., Stumpf S., Zhao H., Errico J.M., Theel E.S., Liebeskind M.J. (2021). Identification of SARS-CoV-2 spike mutations that attenuate monoclonal and serum antibody neutralization. Cell Host Microbe.

[66] Greaney A.J., Starr T.N., Gilchuk P., Zost S.J., Binshtein E., Loes A.N., Hilton S.K., Huddleston J., Eguia R., Crawford K.H. (2021). Complete mapping of mutations to the SARS-CoV-2 spike receptor-binding domain that escape antibody recognition. Cell Host Microbe.

[67] Planas D., Veyer D., Baidaliuk A., Staropoli I., Guivel-Benhassine F., Rajah M.M., Planchais C., Porrot F., Robillard N., Puech J. (2021). Reduced sensitivity of SARS-CoV-2 variant Delta to antibody neutralization. Nature.

[68] McCallum M., Bassi J., Marco A.D., Chen A., Walls A.C., Iulio J.D., Tortorici M.A., Navarro M.-J., Silacci-Fregni C., Saliba C. (2021). SARS-CoV-2 immune evasion by the B.1.427/B.1.429 variant of concern. Science.

[69] Motozono C., Toyoda M., Zahradnik J., Saito A., Nasser H., Tan T.S., Ngare I., Kimura I., Uriu K., Kosugi Y. (2021). SARS-CoV-2 spike L452R variant evades cellular immunity and increases infectivity. Cell Host Microbe.

[70] Starr T.N., Greaney A.J., Dingens A.S., Bloom J.D. (2021). Complete map of SARS-CoV-2 RBD mutations that escape the monoclonal antibody LY-CoV555 and its cocktail with LY-CoV016. Cell Rep. Med..

[71] Meng B., Kemp S.A., Papa G., Datir R., Ferreira I.A., Marelli S., Harvey W.T., Lytras S., Mohamed A., Gallo G. (2021). Recurrent emergence of SARS-CoV-2 spike deletion H69/V70 and its role in the Alpha variant B. 1.1. 7. Cell Rep..

[72] Harvey W.T., Carabelli A.M., Jackson B., Gupta R.K., Thomson E.C., Harrison E.M., Ludden C., Reeve R., Rambaut A., Peacock S.J. (2021). SARS-CoV-2 variants, spike mutations and immune escape. Nat. Rev. Microbiol..

[73] Garcia-Beltran W.F., Lam E.C., Denis K.S., Nitido A.D., Garcia Z.H., Hauser B.M., Feldman J., Pavlovic M.N., Gregory D.J., Poznansky M.C. (2021). Multiple SARS-CoV-2 variants escape neutralization by vaccine-induced humoral immunity. Cell.

[74] Noh J.Y., Jeong H.W., Shin E.-C. (2021). SARS-CoV-2 mutations, vaccines, and immunity: Implication of variants of concern. Signal Transduct. Target. Ther..

[75] Wang P., Nair M.S., Liu L., Iketani S., Luo Y., Guo Y., Wang M., Yu J., Zhang B., Kwong P.D. (2021). Antibody resistance of SARS-CoV-2 variants B. 1.351 and B. 1.1. 7. Nature.

[76] Wang Z., Schmidt F., Weisblum Y., Muecksch F., Barnes C.O., Finkin S., Schaefer-Babajew D., Cipolla M., Gaebler C., Lieberman J.A. (2021). mRNA vaccine-elicited antibodies to SARS-CoV-2 and circulating variants. Nature.

[77] Chen R.E., Zhang X., Case J.B., Winkler E.S., Liu Y., VanBlargan L.A., Liu J., Errico J.M., Xie X., Suryadevara N. (2021). Resistance of SARS-CoV-2 variants to neutralization by monoclonal and serum-derived polyclonal antibodies. Nat. Med..

[78] Ferreira I., Datir R., Papa G., Kemp S., Meng B., Rakshit P., Singh S., Pandey R., Ponnusamy K., Radhakrishnan V.S. (2021). SARS-CoV-2 B. 1.617 emergence and sensitivity to vaccine-elicited antibodies. BioRxiv.

[79] Singh U.B., Rophina M., Chaudhry R., Senthivel V., Bala K., Bhoyar R.C., Jolly B., Jamshed N., Imran M., Gupta R. (2021). Genomic analysis of symptomatic SARS-CoV-2 vaccine breakthrough infections from a tertiary care centre in India. OSF Prepr..

[80] Ikegame S., Siddiquey M.N.A., Hung C.-T., Haas G., Brambilla L., Oguntuyo K.Y., Kowdle S., Chiu H.-P., Stevens C.S., Vilardo A.E. (2021). Neutralizing activity of Sputnik V vaccine sera against SARS-CoV-2 variants. Nat. Commun..

