# Supplementary material for: Circulation and Evolution of SARS-CoV-2 in India: Let the Data Speak
Source: Viruses. 2021 Nov 8;13(11):2238. doi: 10.3390/v13112238 (PMC8619538; doi:10.3390/v13112238)
Supplement: Supplementary file 1 [file viruses-13-02238-s001.zip › Supplementary file 7.pdf]

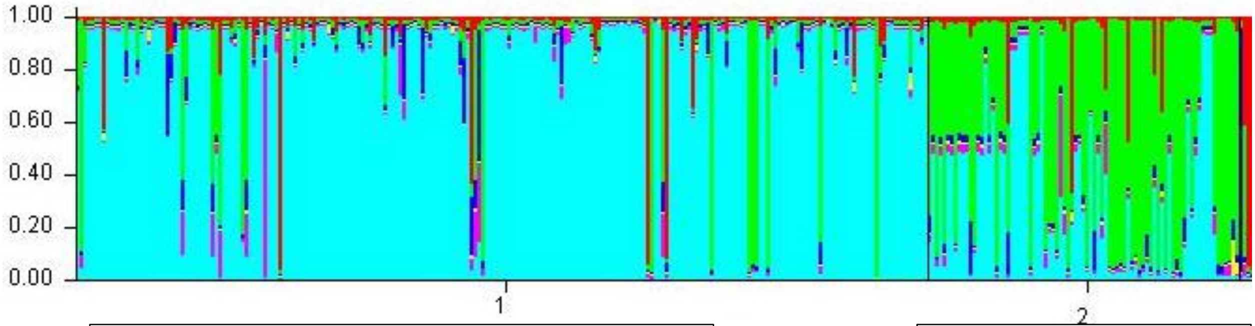

NextClade: 19A; Lineage: Others; Count: 227

NextClade: 19B; Lineage: Others; Count: 83

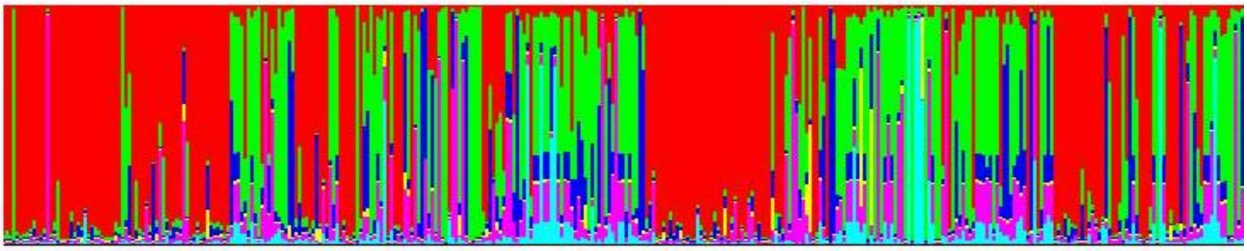

NextClade: 20A; Lineage: VoC-Delta, VoI-Kappa, Eta, B.1.617.3 and Others Count: 2575

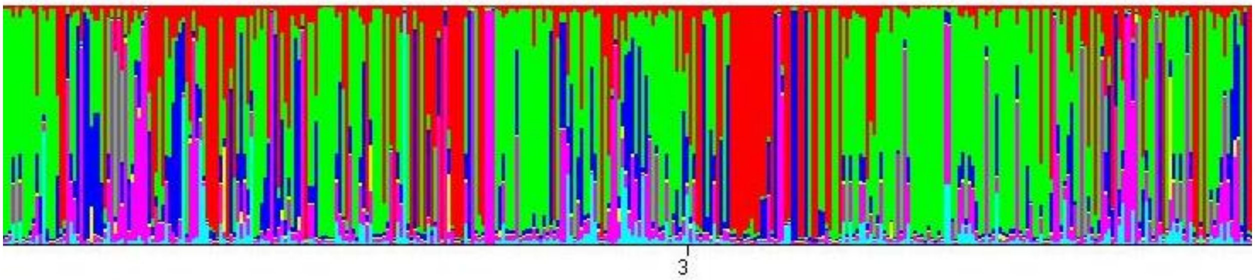

NextClade: 20B; Lineage: VoI-Zeta and Others; Count: 1742

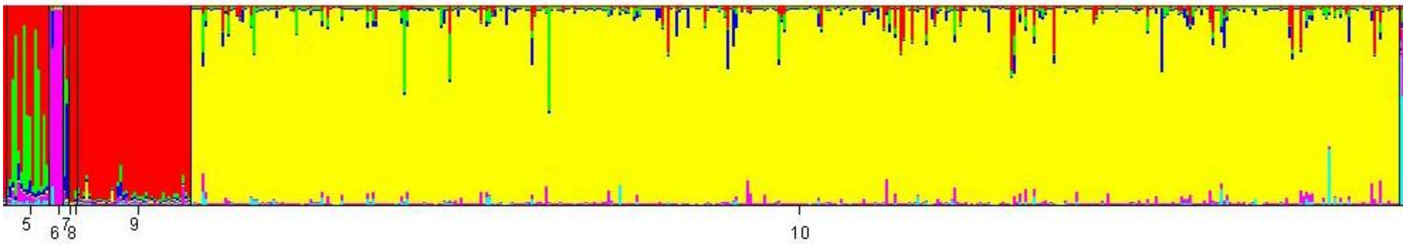

5: NextClade: 20C; Lineage: Others; Count: 15

6: NextClade: 20D; Lineage: Others; Count: 4

7: NextClade: 20E; Lineage: Others; Count: 2

8: NextClade: 20G; Lineage: Others; Count: 8

9: NextClade: 20H/501Y.V2; Lineage: VoC-Beta and Others; Count: 40

NextClade: 20I/501Y.V1; Lineage: VoC-Alpha and Others; Count:

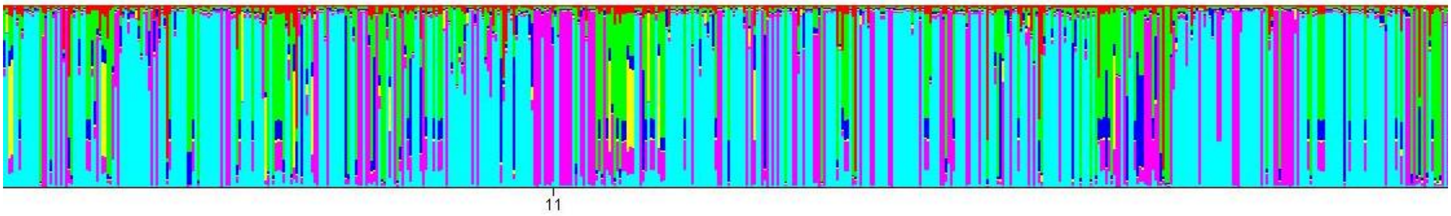

NextClade: 21A; Lineage: VoC- Delta, VoI-Kappa, B.1.617.3 and Others; Count: 1641
