# Supplementary figures and images for: Circulation and Evolution of SARS-CoV-2 in India: Let the Data Speak
Source: Viruses. 2021 Nov 8;13(11):2238. doi: 10.3390/v13112238 (PMC8619538; doi:10.3390/v13112238)

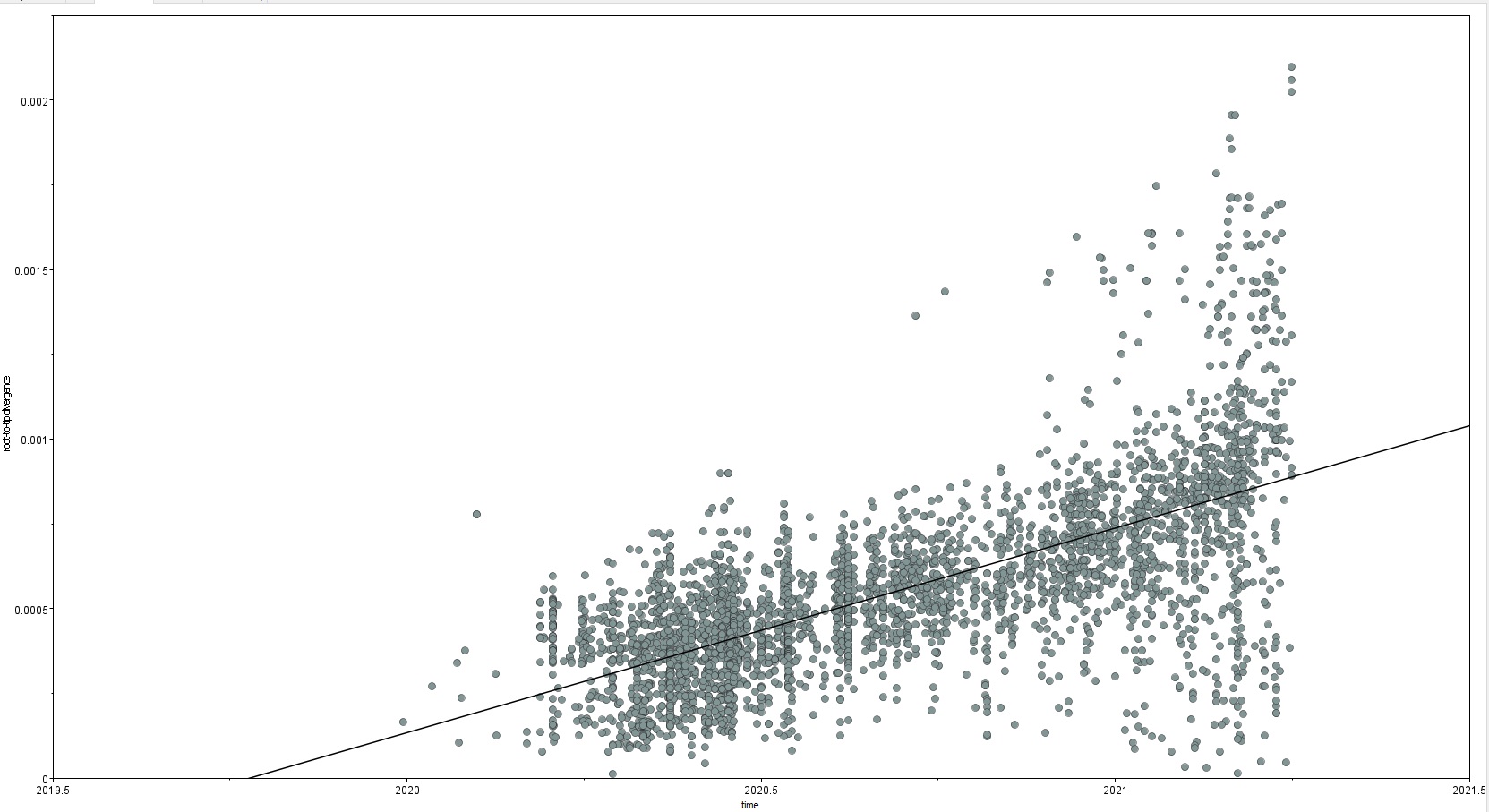

Supplement: Supplementary file 1 [file viruses-13-02238-s001.zip › Supplementary file 6.jpg]
